# Association Study of *CACNA1D*, *KCNJ11*, *KCNQ1*, and *CACNA1E* Single-Nucleotide Polymorphisms with Type 2 Diabetes Mellitus

**DOI:** 10.3390/ijms25179196

**Published:** 2024-08-24

**Authors:** Juan Daniel Díaz-García, Margarita Leyva-Leyva, Fabiola Sánchez-Aguillón, Mercedes Piedad de León-Bautista, Abel Fuentes-Venegas, Alfredo Torres-Viloria, Erika Karina Tenorio-Aguirre, Sara Luz Morales-Lázaro, Angélica Olivo-Díaz, Ricardo González-Ramírez

**Affiliations:** 1 División de Medicina Interna, Hospital General “Dr. Manuel Gea González”, Mexico City 14080, Mexico; judandigar@gmail.com (J.D.D.-G.); fuva409@me.com (A.F.-V.); alfred.torr75@gmail.com (A.T.-V.); karitenorio03@gmail.com (E.K.T.-A.); 2 Departamento de Biología Molecular e Histocompatibilidad, Hospital General “Dr. Manuel Gea González”, Mexico City 14080, Mexico; margaritaleyvaleyva@yahoo.com.mx (M.L.-L.); ofalabi_j@hotmail.com (F.S.-A.); aolivod@yahoo.com (A.O.-D.); 3 Escuela de Medicina, Universidad Vasco de Quiroga, Morelia 58090, Mexico; dramercedespiedad@gmail.com; 4 Laboratorio de Enfermedades Infecciosas y Genómica (INEX LAB), Morelia 58280, Mexico; 5 División de Neurociencias, Instituto de Fisiología Celular, Universidad Nacional Autónoma de México, Mexico City 04510, Mexico; saraluzm@ifc.unam.mx; 6 Centro de Investigación Sobre el Envejecimiento, CINVESTAV, Mexico City 14330, Mexico

**Keywords:** *KCNQ1*, *KCNJ11*, *CACNA1D*, *CACNA1E*, Type 2 diabetes mellitus, single-nucleotide polymorphism (SNP), association study

## Abstract

Type 2 diabetes mellitus (T2DM) is a complex chronic disease characterized by decreased insulin secretion and the development of insulin resistance. Previous genome-wide association studies demonstrated that single-nucleotide polymorphisms (SNPs) present in genes coding for ion channels involved in insulin secretion increase the risk of developing this disease. We determined the association of 16 SNPs found in *CACNA1D*, *KCNQ1*, *KCNJ11*, and *CACNA1E* genes and the increased probability of developing T2DM. In this work, we performed a case-control study in 301 Mexican adults, including 201 cases with diabetes and 100 controls without diabetes. Our findings indicate a moderate association between T2DM and the C allele, and the C/C genotype of rs312480 within *CACNA1D*. The CAG haplotype surprisingly showed a protective effect, whereas the CAC and CGG haplotypes have a strong association with T2DM. The C allele and C/C genotype of rs5219 were significantly associated with diabetes. Also, an association was observed between diabetes and the A allele and the A/A genotype of rs3753737 and rs175338 in *CACNA1E*. The TGG and CGA haplotypes were also found to be significantly associated. The findings of this study indicate that the SNPs examined could serve as a potential diagnostic tool and contribute to the susceptibility of the Mexican population to this disease.

## 1. Introduction

Type 2 diabetes mellitus (T2DM) is a prevalent condition worldwide that requires immediate attention. According to data from the World Health Organization (WHO), the prevalence of T2DM is elevated. Obesity, overweight, and a sedentary lifestyle are risk factors that contribute to a population’s susceptibility to developing T2DM, and these factors are strongly influenced by genetic factors. In Mexico, T2DM is classified as a priority disease in the national health scheme due to its increasing prevalence, and official data show that approximately 18% of the Mexican population has this disease, with a higher incidence in women than in men. T2DM is a prevalent multifactorial disorder that is characterized by low insulin secretion by pancreatic β-cells and impaired insulin action on peripheral tissues (insulin resistance), resulting in elevated blood glucose levels (hyperglycemia). Genes that regulate insulin secretion are plausible candidates for the increased risk of developing T2DM. Genome-wide association studies (GWAS) have successfully identified around 150 genetic loci linked to the risk of T2DM [1]. Furthermore, several genes identified through GWAS are directly involved in insulin secretion by pancreatic β-cells. Studies have demonstrated that genetic mutations in *TCF7L2*, *KCNJ11*, *TCF14*, *TCF1*, *HHEX*, *CDKAL1*, *CDKN1A/B*, and *IGF2BP2* can affect insulin secretion and increase susceptibility to T2DM [2,3,4,5,6,7,8,9].

Pancreatic β-cells have multiple ion channels within their plasma membranes and subcellular organelles. The regulation of glucose-dependent insulin secretion is influenced by the coordinated activity and sensitivity of these ion channels [1]. We will focus on SNPs of potassium- and voltage-dependent calcium channels involved in insulin secretion. In multiple populations [10], including the Mexican population [11], numerous studies have been conducted to ascertain the presence of SNPs related to T2DM. One gene that has been extensively researched is *KCNQ1*. *KCNQ1* is responsible for encoding the voltage-gated potassium channel subfamily Q member 1 (K_ir_7.1), and this gene is expressed in both the human heart and pancreas. In two separate GWAS, *KCNQ1* was initially identified as a gene associated with susceptibility to T2DM in populations of East Asian descent [10,12]. Subsequently, several studies have confirmed that *KCNQ1* is a susceptibility gene for T2DM in Chinese, Singaporean, Indian, and Euro-Caucasian populations. Other studies have demonstrated an association between the SNPs rs2237892, rs2237895, rs2237897, rs2283228, and rs163184 with T2DM. These SNPs exhibit variable allele frequencies, from 30–40% in Asians, while in Europeans they are only 10% [13,14]. Functional studies conducted on *KCNQ1* have shown that insulin secretion can be stimulated by selectively blocking K^+^ channels. Additionally, examination of clinical characteristics indicated that individuals carrying the *KCNQ1* risk allele exhibited compromised insulin secretion at baseline.

K_ATP_ is another potassium channel that is implicated in insulin secretion. *KCNJ11* is located at 11p15.1 and encodes potassium inwardly rectifying channel subfamily J member 11 (K_ir_6.2). The K_ir_6.2 channel and sulfonylurea receptor 1 (SUR1) form a complex that gives rise to the K_ATP_ channel [15]. Knockout mice lacking this specific channel show impairments in their capacity to secrete insulin in response to glucose stimulation [16]. A significant association has been reported between the allelic variant E23K (G→A, rs5219) of *KCNJ11* and the occurrence of T2DM in various ethnic populations, such as French Caucasian and Japanese populations [17,18,19]. The E23K (G→A, rs5219) and A190A (C→T, rs5218) polymorphisms were examined in a Chinese population to determine their correlation with early-onset T2DM (<40 years) and hypertension. The results of that study indicated that individuals carrying the E23K-GA or AA genotypes may have an increased susceptibility to the development of early-onset T2DM. In contrast, genotypes A190A-TT or E23K-GG may potentially increase susceptibility to hypertension [20].

Human genetic studies have shown that *CACNA1D* is the most prominent gene associated with T2DM. *CACNA1D* is located on chromosome 3p21.1. and encodes Ca_V_1.3. Studies conducted on Scandinavian populations have successfully identified three SNPs associated with T2DM and changes in insulin secretion. The C allele of rs312480 was linked to T2DM and a decrease in insulin secretion by pancreatic β-cells. On the other hand, the G allele of rs312486 and rs9841978 exhibited a significant association solely with T2DM [21]. Ca_V_2.3, which is encoded by the *CACNA1E* gene located on chromosome 1q25.3, is expressed in pancreatic β-cells [22] and plays an essential role in the release of insulin [23]. Silencing the expression of Ca_V_2.3 in INS-1 cells reduces insulin secretion stimulated by glucose [24].

Additionally, knockout mice exhibit fasting hyperglycemia and impaired glucose tolerance [25,26]. Earlier studies conducted in two distinct ethnic groups have documented an association between *CACNA1E* variants and the risk of developing T2DM. The initial investigation involved sequencing of the *CACNA1E* gene in Pima Indians, which revealed that five variants (rs625226, rs3753737, rs798209, rs473200, and +8130 G/A) were significantly associated with the early onset of T2DM. The SNPs rs3753737 and +8130 G/A were significantly associated with T2DM in a family analysis. The G allele of the +8130 G/A variant, located in the 3′-UTR region, is associated with the early onset of T2DM. Among 372 Pima individuals without diabetes who participated in metabolic testing, the presence of the risk allele (G allele of +8130 G/A) was found to be correlated with diminished insulin action. This was evidenced by elevated levels of fasting plasma glucose and insulin concentrations observed during an oral glucose tolerance test [27,28,29]. The second study, performed on Italian individuals who were recently diagnosed with T2DM, identified ten distinct SNPs that encompassed 93% of the usual variation in *CACNA1E*. These SNPs include rs558994, rs679931, rs2184945, rs10797728, rs3905011, rs12071300, rs175338, rs3753737, rs2253388, and rs4652679. Five SNPs (rs10797728, rs175338, rs2184945, rs3905011, and rs4652679) are associated with specific aspects of β-cell function [30].

Given the regulatory role of ion channels in insulin secretion, the objective of this study was to investigate the correlation between T2DM, and 16 SNPs found in *CACNA1D*, *KCNQ1*, *KCNJ11*, and *CACNA1E*. This study was conducted on a sample of 301 unrelated Mexican individuals residing in Mexico City. These findings will enable us to ascertain the potential of previously identified SNPs in predicting T2DM in the Mexican population.

## 2. Results

### 2.1. Biochemical and Medical Characteristics of the Participants in the Study

This study included 301 participants, consisting of 100 individuals classified as healthy controls and 201 individuals diagnosed with T2DM. Table 1 shows the demographic and clinical characteristics of the participants involved in the study. The control group consisted of 52 females and 48 males, with a mean age of 56.184 ± 13.65. The case group, on the other hand, comprised 75 females and 126 males, with a mean age of 46.54 ± 15.14. A notable disparity was observed in the percentage of participants with hypertension between the case (46 individuals) and control (24 individuals) groups. Our findings demonstrated a statistically significant (*p* < 0.001) increase in weight, body mass index (BMI), Hb1AC, and fasting plasma glucose level (Table 1).

### 2.2. Analysis of the Association between SNPs and T2DM

Sixteen polymorphisms in four genes were investigated (Supplementary Table S1). We eliminated rs12487452, rs2283171, rs163184, and rs10797728 for having a Hardy-Weinberg equilibrium *p*-value < 0.05 (Supplementary Table S2). The allele and genotype frequencies for the 12 SNPs in the candidate genes are presented in Table 2 and Table 3, respectively. Regarding the polymorphisms investigated in the *CACNA1D* gene, it was found that the C allele of rs312480 (OR = 2.82; 95% CI 1.22–6.67; *p* = 0.006), C/C genotype of rs312480 (OR = 3.0; 95% CI 1.26–7.25; *p* = 0.005), and C/G genotype of rs312486 (OR = 1.88; 95% CI 0.98–3.74; *p* = 0.03) exhibited a moderate association with susceptibility to T2DM. Concerning the *KCNQ1* gene, none of the alleles or genotypes were significantly associated with T2DM. Analysis of polymorphisms in the *KCNJ11* gene revealed a significant association between the C allele (OR = 1.96; 95% CI 1.35–2.82; *p* = 0.0001) and C/C genotype (OR = 3.79; 95% CI 2.12–6.96; *p* < 0.0001) of rs5219 and the disease. Finally, the A alleles of rs3753737 (OR = 1.66; 95% CI 1.16–2.38; *p* = 0.002) and rs175338 (OR = 1.65; 95% CI 1.11–2.48; *p* = 0.005) in the *CACNA1E* gene were associated with increased susceptibility to the development of T2DM. Additionally, the genotypes A/A of rs3753737 (OR = 1.79; 95% CI 0.99–3.33; *p* = 0.02) and rs175338 (OR = 4.59; 95% CI 1.34–24.2; *p* = 0.004) were significantly associated with diabetes.

### 2.3. Model of Inheritance Analysis

Table 4 shows the data pertaining to the codominant, dominant, and recessive inheritance models for the SNPs. The rs312480 polymorphism of *CACNA1D* was not included in the analysis because of the limited number of genotypes (C/C and C/T, as shown in Table 3). The selection of the best model for each SNP was based on Akaike’s Information Criterion (AIC) and the Bayesian Information Criterion (BIC), with a preference given to those with the lowest score [31]. The co-dominant and dominant models for rs312486 in *CACNA1D* showed a protective effect against T2DM (OR = 0.41; 95% CI 0.19–0.89; *p* = 0.036 and OR = 0.44; 95% CI 0.21–0.95; *p* = 0.03, respectively). For SNPs in *KCNQ1*, codominant and dominant patterns of rs2237897, rs2283228, and rs2237892 were determined to be associated with T2DM. The rs175338 polymorphism in *CACNA1E* had a protective effect against diabetes in the three models examined. In the codominant model, the OR was 0.62 (95% CI: 0.33–1.17; *p* < 0.0001); in the dominant model, it was 0.46 (95% CI: 0.25–0.83; *p* = 0.009); and in the recessive model, it was 0.09 (95% CI: 0.02–0.38; *p* < 0.0001). However, the recessive model fit better, considering the AIC and BIC coefficients. The remaining SNPs did not show any significant association with T2DM (*p* > 0.05).

### 2.4. Association of Haplotypes with T2DM

The linkage disequilibrium (LD) plot presented in Supplementary Figure S1 illustrates the pairwise values of LD within our study population, which comprised both control individuals and patients (case group). Genotypes that met the criteria of 75% acceptance, a minor allele frequency of 0.01, and a Hardy-Weinberg equilibrium *p*-value <0.05 were included. The rs12487452, rs2283171, rs163184 and rs10797728 polymorphisms were excluded from the analysis (Supplementary Table S2). Consequently, LD analysis was conducted on the remaining 12 SNPs. We employed the confidence interval method to conduct LD analysis. We observed elevated levels of LD in genomic regions associated with the *KCNQ1* and *KCNJ11* genes. For the remaining two genes, *CACNA1D* and *CACNA1E*, the level of LD was lower than that in blocks one and two. Based on the aforementioned observations, our study aimed to investigate the potential correlations between T2DM and the haplotypes associated with the genes under investigation. Table 5 presents the results. The haplotypes CAC (OR = 48.69; CI 95% 12.14–195.25, *p* < 0.0001) and CGG (OR = 40.77; CI 95% 5.00–332.33, *p* = 0.000004) of the *CACNA1D* gene exhibited a strong association with diabetes, while the CAG haplotype showed a protective effect against the disease. The TTCT haplotype in *KCNQ1* was moderately associated with T2DM (OR = 1.8; 95% CI 1.13–3.04; *p* = 0.015). In the *CACNA1E* gene, the TGG (OR = 7.14; 95% CI 2.55–19.98; *p* = 0.0002) and CGA (OR = 13.360; 95% CI 1.45–123.23; *p* = 0.023) haplotypes demonstrated an association with the development of T2DM. *KCNJ11* haplotypes were not directly related to diabetes.

### 2.5. Gene-Interaction Network

**Figure 1:**
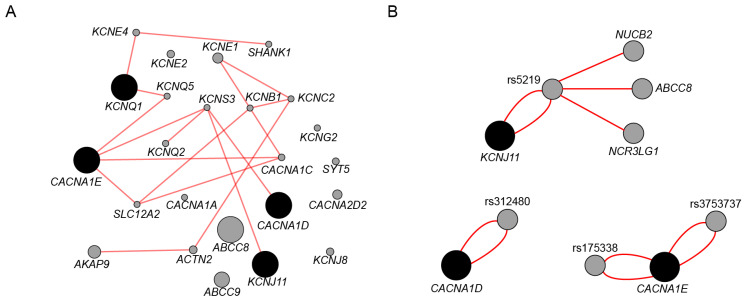
Gene-interaction analysis for *CACNA1D*, *KCNJ11*, and *CACNA1E*. (**A**) Analysis of the four genes (represented by black circles) using the bioinformatics program GeneMANIA. The red lines illustrate the interplay between various genes. (**B**). Interactions of SNPs associated with T2DM are shown.

T2DM is a complex condition characterized by the involvement of multiple genes. To determine potential interactions between the genes investigated in this study and other genes, the GeneMANIA tool was employed. GeneMANIA defines a genetic interaction between two genes as the occurrence of a change in one gene that influences the behavior or function of the other gene [32]. Figure 1A presents the findings of the analysis, demonstrating that the four genes under investigation interacted with a diverse range of genes. The majority of these interacting genes are other ion channels, including *CACNA1C*, which encodes the Ca_V_1.2 channel. Additionally, there are genes encoding auxiliary subunits associated with potassium channels, such as *KCNB1*, which encodes the potassium voltage-activated channel subfamily M regulatory beta subunit 1. Surprisingly, none of the four genes exhibited direct interactions. Additionally, it is important to note that the *ABCC8* and *KCNJ11* genes, despite jointly forming K_ATP_, do not directly interact with each other.

To augment the gene-interaction network, the 3DSNP database was employed. This database serves as a tool for SNP annotation, providing information on the three-dimensional interactions of SNPs with other genes, and assigning them a functionality score. We conducted an analysis of a specific set of SNPs that have been previously linked to T2DM. These SNPs were rs312480 (*CACNA1D*), rs5219 (*KCNJ11*), and rs3753737–rs175338 (*CACNA1E*). Figure 1B presents a summary of the obtained results, indicating that SNPs in the *CACNA1D* and *CACNA1E* genes did not exhibit any interactions with other genes, but only with themselves. The rs5219 was found to be in contact with two genes, *NUCB2* and *ABCC8*, which are associated with diabetes. Interestingly, this SNP interacts with *ABCC8*, a gene responsible for encoding SUR1.

## 3. Discussion

Diabetes is the third most prevalent cause of mortality in Mexico. Therefore, it is imperative to enhance research on genetic factors that can function as markers for predicting the onset of this disease. These markers could aid in the implementation of preventive measures. T2DM is a polygenic disorder influenced by multiple genetic mutations that contribute to an increased susceptibility to the disease in certain individuals [1]. Regulation of insulin secretion is intricately governed by the activity of multiple ion channels. Any malfunction or improper expression of genes responsible for these channels can lead to a reduction in insulin secretion, ultimately resulting in the development of hyperglycemia.

Previous research conducted on non-Mexican populations has demonstrated that genetic variations in the *CACNA1D*, *KCNQ1*, *KCNJ11*, and *CACNA1E* genes are associated with an elevated susceptibility to developing T2DM. Hence, considering the existence of ethnic variations among populations, the objective of this study was to investigate the potential correlation between polymorphisms found in the genes responsible for encoding these ion channels and the diagnosis of T2DM. This study is significant as it is the first comprehensive examination of SNPs in these ion channels in Latin American and Mexican populations.

SNPs located in *KCNQ1*, specifically rs2237897, rs2283228, and rs163184, have been found to be significantly associated with T2DM. Previous studies [13,14] have reported varying allele frequencies for these SNPs. In Asian populations, the allele frequencies range from 30% to 40%, whereas in European populations, the frequencies are only approximately 10%. Interestingly, our study revealed that the allele frequencies of rs2237897 and rs2283228 in the Mexican populations were 55–57%. This frequency is approximately twice as high as that observed in the Asian population and five to six times higher than that observed in the European population. Surprisingly, SNPs in *KCNQ1* were not associated with diabetes in our population, which represents a major difference from studies in other populations.

In the *CACNA1D* gene, an association was observed between the C allele of rs312480, the C/C genotype of rs312480, and the C/G genotype of rs312486 with T2DM. Interestingly, the CAC and CGG haplotypes exhibited a strong association with T2DM, unlike the CAG haplotype, which has a protective effect. Regrettably, existing literature lacks data to compare and contrast our findings. These SNP have been identified in Scandinavian populations as risk factors associated with T2DM and are known to induce changes in insulin secretion [21]. *CACANA1D* single SNPs have garnered limited international interest, with the analysis being restricted to the Scandinavian population. In light of this rationale, we posit that, in conjunction with our findings, these outcomes will establish a benchmark for conducting more comprehensive investigations across diverse populations, aiming to identify a potential correlation with T2DM.

Regarding the *CACNA1E* SNPs, the existing literature has reported a link between these SNPs and T2DM in different populations, including Europeans, East Asians, and African Americans [33,34,35,36]. A study conducted among the Pima Indians, an indigenous population residing in Arizona (United States) and, to a lesser extent, in Sonora and Chihuahua (Mexico), revealed that *CACNA1E* plays a role in the susceptibility to T2DM by influencing insulin action [22,24]. In the present study, our findings align with those of previous research conducted on Pima Indians, as we observed a significant association between the A allele and A/A genotype of rs3753737 and rs175338 with the development of T2DM.

Our findings revealed a significant correlation between T2DM and the presence of the C allele as well as the C/C genotype of the rs5219 variant situated within the *KCNJ11* gene. Our results are consistent with data obtained in other populations, where a similar association was observed among various ethnic groups, including French Caucasians, Japanese, and Chinese [17,18,20].

Finally, we showed that *CACNA1D*, *KCNQ1*, *KCNJ11*, and *CACNA1E* are part of a gene-interaction network. Although not directly linked, they interact with multiple genes associated with diabetes, obesity, and insulin secretion. Among the genes that exhibit interaction with *CACNA1E*, the *SLC12A2* gene, which encodes the solute carrier family 12 (sodium/potassium/chloride transporter) member 2, has been recently associated to metabolic syndrome in mice [37]. The *KCNQ5* gene encoding the K_V_7.5 channel is linked to the susceptibility to obesity in the Korean population [38]. Our results indicate that it is associated with both the *CACNA1E* and *KCNQ1* genes. An intriguing finding revealed that *KCNJ11* does not directly interact with *ABCC8*. However, it has been observed that SNP rs5219 establishes physical contact with *ABCC8*. This interaction may play a role in the regulation of SUR1 expression. This interaction network provides evidence for the existence of various factors that govern the function of pancreatic β-cells and contribute to the development of T2DM. Consequently, these factors may affect the synthesis and secretion of insulin. Our study has some limitations, among which are the low sample size, the difference in the number of individuals between the two groups, and the lack of clinical data in the control group; however, we consider that this study evidences the importance of the SNPs studied and the predisposition to develop T2DM in the Mexican population.

## 4. Materials and Methods

### 4.1. Study Population

A case–control study was conducted involving 301 unrelated Mexican adults aged between 30 and 90 years. This study included 201 patients who had been diagnosed with T2DM for at least one year according to the clinical criteria established by the American Diabetes Association. These criteria included fasting glucose levels exceeding 6.99 mmol/L, random glucose levels exceeding 11.1 mmol/L on at least two occasions, oral glucose tolerance levels of 11.1 mmol/L or higher, and HbA1c levels exceeding 6.5%. The control group consisted of 100 individuals who were not diagnosed with T2DM. This study incorporated specific exclusion criteria, which encompassed clinically significant liver, thyroid, or rheumatologic diseases as well as documented chronic conditions such as cancer. The population enrollment was performed at the Department of Internal Medicine of the Hospital General Dr. Manuel Gea González.

### 4.2. DNA Isolation and Genotyping

Genomic DNA was isolated from a 10 mL sample of EDTA-treated peripheral blood using Proteinase K and the phenol–chloroform protocol [39]. The concentration of extracted DNA was determined using a NanoDrop One Spectrophotometer (Thermo Fisher Scientific, Waltham, MA, USA). Sixteen polymorphisms located in four genes known to be associated with T2DM (Supplementary Table S1) were chosen for genotyping. Genotyping was performed using MALDI-TOF mass spectrometry iPLEX technology with a MassArray System (Agena Bioscience Inc., San Diego, CA, USA). PCR and iPLEX extension primers were designed based on sequences containing each target SNP. The Assay Design Suite version 2.0 software was used with default settings for primer design. Single-base extension reactions were performed on the PCR reactions using the iPLEX Gold Kit (AgenaBioscience., San Diego, CA, USA). To ensure consistency and precision, a random subset comprising 10% of samples was subjected to repeated genotyping. The results showed a 100% agreement.

### 4.3. Statistical Analysis

The clinical characteristics of the groups diagnosed with T2DM and the control group were subjected to statistical analysis using the Mann-Whitney Rank Sum Test since they failed the normality test. The analyses were performed using GNU PSPP 2.0.0-pre1 software (Software Foundation. Boston, MA, USA). Genotyping data were analyzed using the PLINK 1.9 software [40]. Subsequently, a case–control association analysis was performed on the available samples. Genotype frequencies were evaluated for adherence to the Hardy-Weinberg equilibrium (HWE) using the goodness-of-fit chi-squared test (Supplementary Table S2). The rs12487452, rs2283171, rs163184, and rs10797728 polymorphisms were eliminated from the analysis because they did not meet the HWE. The odds ratio (OR) and 95% confidence intervals (95% CIs) were calculated using the chi-squared with a Fisher exact test logistic regression. These calculations were performed using the PLINK 1.9 program and corroborated with the StatCalc tool of the Epi Info 7 software [41]. Haplotypes and linkage disequilibrium (LD) blocks were generated using the CI method and analyzed with the Haploview 4.2 software. Haplotypes and LD blocks were generated using the CI method and analyzed with the Haploview 4.2 software [42]. The PLINK 1.9 and SNPStats programs [31] were used to determine the most informative Mendelian association model (codominant, dominant, and recessive). Akaike’s Information Criterion (AIC) and the Bayesian Information Criterion (BIC) were used to ascertain the optimal model [31]. Bonferroni adjustment for multiple comparisons correction with final *p* values of ≤0.004 were considered statistically significant according to the equation: *p* value 0.05/12 = 0.004 (as we studied 12 SNPs), and those between 0.05 and 0.004 were regarded as moderately significant.

### 4.4. Bioinformatic Analysis

We used the GeneMANIA tool "http://www.genemania.org (accessed on 12 august 2024)" to generate a gene-interaction network for the *CACNA1D*, *KCNQ1*, *KCNJ11*, and *CACNA1E* genes. GeneMANIA utilizes diverse datasets and resources to forecast genes that exhibit strong interactions with a specific gene [32]. We also employed the 3DSNP tool "https://omic.tech/3dsnpv2 (accessed on 14 august 2024)", a computational tool that predicts the three-dimensional interactions between SNPs and other genes [43]. The Cytoscape program [44] was employed to construct the polymorphism-interaction network by utilizing data acquired from the 3DSNP database.

## 5. Conclusions

Our study provides evidence of a correlation between well-established genetic variants of *CACNA1D*, *KCNQ1*, *KCNJ11*, and *CACNA1E* and T2DM in the Mexican population. These SNPs may serve as a diagnostic tool for this disease and are considered predisposing factors owing to their high frequency and strong association observed in our study subjects. These data indicate the strong susceptibility of our population to the development of T2DM in the presence of these SNPs. However, it is important to note that the behavior of these SNPs may vary among different populations, thereby emphasizing the existence of variation and significant ethnic heterogeneity among individuals. The investigation of SNPs linked to T2DM has gained momentum. We assert that the identification of a panel of polymorphisms is essential for their potential utilization as diagnostic and therapeutic tools in the early detection and management of this condition. Furthermore, this approach enables exploration of pharmacogenetic and pharmacogenomic strategies to address this disease.

## Figures and Tables

**Table 1:** Distribution of baseline clinical and biochemical characteristics of the studied population.

Variables	Case Group *n* = 201 Mean ± SD	Control Group *n* = 100 Mean ± SD	*p*
Females/males ^ϯ^	75/126	52/48	n/a
Age (years)	56.184 ± 13.65	46.540 ± 15.143	<0.001 *
Duration of disease (years)	13.597 ± 5.246	n/a	n/a
Weight (kg)	73.5 ± 13.6	66.480 ± 6.790	<0.001 *
BMI (kg/m^2^)	26.77 ± 4.14	23.343 ± 2.171	<0.001 *
HTN (%)	46	24	<0.001 *
Hb1AC (%)	8.562 ± 2.27	4.871 ± 0.264	<0.001 *
FPG (mmol/L)	8.0 ± 1.7	4.63 ± 0.39	<0.001 *

*n* = total sample size, BMI = body mass index, HTN = hypertension, Hb1AC = glycosylated hemoglobin, FPG = fasting plasma glucose, n/a = not applicable. * *p* value with Mann-Whitney rank sum test, significance level at *p* ≤ 0.05. ^ϯ^ For this variable, the ratio of females/males is presented instead of the mean ± SD.

**Table 2:** Allele frequencies of *CACNA1D*, *KCNQ1*, *KCNJ11*, and *CACNA1E* single-nucleotide polymorphisms (SNPs) in Type 2 diabetes mellitus (T2DM) patients and healthy controls. Data were adjusted for the covariates sex, age, and BMI.

Gene	SNP	Allele	Case/Control Frequencies		*p*	OR (95% CI)
*CACNA1D*	**rs312480**	**T**	**0.03**	**0.08**	**0.006 ^ϯ^**	**0.35 (0.14–0.81)**
		**C**	**0.97**	**0.92**		**2.82 (1.22–6.67)**
	rs9841978	G	0.84	0.8	0.20	1.23 (0.77–1.91)
		A	0.16	0.2		0.81 (0.51–1.29)
	rs312486	G	0.13	0.1	0.16	1.36 (0.77–2.49)
		C	0.87	0.9		0.73 (0.40–1.29)
*KCNQ1*	rs2237897	C	0.7	0.7	0.50	0.98 (0.66–1.44)
		T	0.3	0.3		1.01 (0.69–1.50)
	rs2074196	G	0.67	0.69	0.38	0.92 (0.63–1.35)
		T	0.33	0.31		1.07 (0.73–1.55)
	rs2283228	A	0.68	0.69	0.45	0.96 (0.65–1.40)
		C	0.32	0.31		1.03 (0.71–1.52)
	rs2237892	C	0.7	0.7	0.45	0.96 (0.64–1.41)
		T	0.3	0.3		1.04 (0.70–1.53)
*KCNJ11*	rs5218	G	0.93	0.94	0.44	0.89 (0.41–1.82)
		A	0.07	0.06		1.11 (0.54–2.40)
	rs5219	**T**	**0.3**	**0.46**	0.0001 *	**0.50 (0.35–0.73)**
		**C**	**0.7**	**0.55**		**1.96 (1.35–2.82)**
*CACNA1E*	rs2253388	T	0.37	0.36	0.43	1.04 (0.72–1.51)
		C	0.63	0.64		0.88 (0.61–1.28)
	rs3753737	**G**	**0.42**	**0.55**	0.002 *	**0.59 (0.41–0.85)**
		**A**	**0.58**	**0.45**		**1.66 (1.16–2.38)**
	rs175338	**A**	**0.34**	**0.24**	**0.005 ^ϯ^**	**1.65 (1.11–2.48)**
		**G**	**0.66**	**0.76**		**0.60 (0.40–0.90)**

Bold characters indicate association. OR (95% CI) = odds ratio (95% confidence interval). * Strong significance level at *p* ≤ 0.004 (after Bonferroni correction) and ^ϯ^ moderate significance level at *p* between 0.05 and 0.004.

**Table 3:** Genotype frequency distribution of *CACNA1D*, *KCNQ1*, *KCNJ11*, and *CACNA1E* in T2DM patients and healthy controls. Data were adjusted for the covariates sex, age, and BMI.

Gene	SNP-Genotypes		Case/Control Frequencies		*p*	OR (95%CI)
*CACNA1D*	rs312480	C/T	0.06	0.16	0.26	0.33 (0.13–0.79)
		**C/C**	**0.94**	**0.84**	**0.005 ^ϯ^**	**3.0 (1.26–7.25)**
	rs9841978	G/G	0.7	0.67	0.33	1.15 (0.66–1.99)
		G/A	0.27	0.27	0.54	0.99 (0.56–1.78)
		A/A	0.03	0.06	0.17	0.48 (0.12–1.86)
	rs312486	G/G	0	0.02	n/a	n/a
		**C/G**	**0.26**	**0.16**	**0.03 ^ϯ^**	**1.88 (0.98–3.74)**
		C/C	0.74	0.82	0.06	0.61 (0.31–1.14)
*KCNQ1*	rs2237897	C/C	0.51	0.49	0.40	1.09 (0.65–1.81)
		C/T	0.37	0.42	0.22	0.80 (0.47–1.35)
		T/T	0.12	0.09	0.28	1.37 (0.58–3.49)
	rs2074196	G/G	0.46	0.47	0.50	0.97 (0.58–1.61)
		G/T	0.42	0.44	0.43	0.93 (0.55–1.56)
		T/T	0.12	0.09	0.33	1.30 (0.53–3.34)
	rs2283228	A/A	0.48	0.47	0.46	1.05 (0.63–1.75)
		A/C	0.4	0.44	0.28	0.84 (0.50–1.41)
		C/C	0.12	0.09	0.28	1.37 (0.58–3.49)
	rs2237892	C/C	0.51	0.5	0.46	1.05 (0.63–1.74)
		C/T	0.37	0.41	0.28	0.83 (0.49–1.41)
		T/T	0.12	0.09	0.28	1.37 (0.58–3.49)
*KCNJ11*	rs5218	G/G	0.87	0.87	0.53	0.96 (0.43–2.04)
		G/A	0.12	0.13	0.51	0.95 (0.44–2.12)
		A/A	0.01	0	n/a	n/a
	rs5219	T/T	0.1	0.12	0.35	0.81 (0.35–1.90)
		**C/T**	**0.4**	**0.67**	<0.0001 *	**0.35 (0.19–0.55)**
		**C/C**	**0.5**	**0.21**	<0.0001 *	**3.79 (2.12–6.96)**
*CACNA1E*	rs2253388	T/T	0.12	0.09	0.24	1.43 (0.61–3.64)
		C/T	0.49	0.54	0.25	0.82 (0.49–1.37)
		C/C	0.38	0.37	0.46	1.05 (0.62–1.79)
	rs3753737	**G/G**	**0.17**	**0.31**	0.004 *	**0.45 (0.24–0.82)**
		A/G	0.51	0.48	0.37	1.11 (0.67–1.85)
		**A/A**	**0.32**	**0.21**	**0.02 ^ϯ^**	**1.79 (0.99–3.33)**
	rs175338	**A/A**	**0.12**	**0.03**	0.004 *	**4.59 (1.34–24.2)**
		G/A	0.44	0.42	0.43	1.07 (0.64–1.80)
		**G/G**	**0.44**	**0.55**	**0.04 ^ϯ^**	**0.63 (0.38–1.06)**

Bold characters indicate association. OR (95% CI) = odds ratio (95% confidence interval), n/a = not applicable. * Strong significance level at *p* ≤ 0.004 (after Bonferroni correction) and ^ϯ^ moderate significance level at *p* between 0.05 and 0.004.

**Table 4:** Effects of *CACNA1D*, *KCNQ1*, *KCNJ11*, and *CACNA1E* on the susceptibility for T2DM under three genetic models. Data were adjusted for the covariates sex, age, and BMI.

Gene	SNP	Model		*p*	OR (95% CI)	AIC/BIC
*CACNA1D*	rs9841978	Codominant	G/G G/A A/A	0.8	1.00 0.85 (0.43–1.71) 1.40 (0.30–6.42)	29/319
		Dominant	G/G G/A-A/A	0.78	1.00 0.91 (0.47–1.76)	295/314
		Recessive	G/G-G/A A/A	0.62	1.00 1.46 (0.32–6.62)	295/314
	rs312486	**Codominant**	**C/C** **C/G** **G/G**	**0.036 ^ϯ^**	**1.00** **0.41 (0.19–0.89)** **n/a**	**291/313**
		**Dominant**	**C/C** **C/G-G/G**	**0.03 ^ϯ^**	**1.00** **0.44 (0.21–0.95)**	**291/309**
		Recessive	C/C-C/G G/G	n/a	1.00 n/a	n/a
*KCNQ1*	rs2237897	**Codominant**	**C/C** **C/T** **T/T**	0.003 *	**1.00** **2.93 (1.52–5.65)** **2.56 (0.83–7.89)**	**286/308**
		**Dominant**	**C/C** **C/T-T/T**	0.0007 *	**1.00** **2.86 (1.53–5.37)**	**284/302**
		Recessive	C/C-C/T T/T	0.44	1.00 1.52 (0.53–4.33)	295/313
	rs2074196	Codominant	G/G G/T T/T	0.12	1.00 1.79 (0.97–3.32) 2.15 (0.70–6.58)	293/315
		**Dominant**	**G/G** **G/T-T/T**	**0.04 ^ϯ^**	**1.00** **1.84 (1.01–3.33)**	**291/310**
		Recessive	G/G-G/T T/T	0.39	1.00 1.60 (0.55–4.63)	295/313
	rs2283228	**Codominant**	**A/A** **C/A** **C/C**	**0.007 ^ϯ^**	**1.0** **2.65 (1.39–5.05)** **2.49 (0.81–7.71)**	**288/310**
		**Dominant**	**A/A** **C/A-C/C**	0.001 *	**1.00** **2.62 (1.41–4.88)**	**286/304**
		Recessive	A/A-C/A C/C	0.44	1.00 1.52 (0.53–4.33)	295/313
	rs2237892	**Codominant**	**C/C** **C/T** **T/T**	**0.007 ^ϯ^**	**1.00** **2.67 (1.39–5.12)** **2.43 (0.79–7.45)**	**287/310**
		**Dominant**	**C/C** **C/T-T/T**	0.001 *	**1.00** **2.63 (1.41–7.45)**	**286/310**
		Recessive	C/C-C/T T/T	0.44	1.00 1.52 (0.53–4.33)	295/313
*KCNJ11*	rs5218	Codominant	G/GG/A A/A	0.39	1.00 0.95 (0.42–2.18) n/a	295/318
		Dominant	G/G G/A-A/A	0.72	1.00 0.86 (0.38–1.94)	295/314
		Recessive	G/G-G/A A/A	0.17	1.00 n/a	293/314
	rs5219	Codominant	C/C T/C T/T	0.16	1.00 1.68 (0.84–3.36) 2.34 (0.88–6.26)	294/316
		Dominant	C/C T/C-T/T	0.07	1.0 1.81 (0.94–3.49)	**292/311**
		Recessive	C/C-T/C T/T	0.23	1.00 1.75 (0.71–4.32)	294/312
*CACNA1E*	rs2253388	Codominant	C/C C/T T/T	0.18	1.00 0.76(0.40–1.43) 0.4 (0.15–1.09)	294/316
		Dominant	C/C C/T-T/T	0.21	1.00 0.68 (0.37–1.25)	294/312
		Recessive	C/C-C/T T/T	0.10	1.00 0.48 (0.19–1.19)	293/311
	rs3753737	Codominant	A/A G/A G/G	0.29	1.00 1.53 (0.75–3.12) 1.84 (0.83–4.07)	295/317
		Dominant	A/A G/A-G/G	0.14	1.0 1.64 (0.84–3.18)	293/312
		Recessive	A/A-G/A G/G	0.3	1.00 1.40 (0.74–2.66)	294/313
	rs175338	**Codominant**	**G/G** **G/A** **A/A**	<0.0001 *	**1.00** **0.62 (0.33–1.17)** **0.08 (0.02–0.31)**	**279/301**
		**Dominant**	**G/G** **G/A-A/A**	**0.009 ^ϯ^**	**1.00** **0.46 (0.25–0.83)**	**289/307**
		**Recessive**	**G/G-G/A** **A/A**	<0.0001 *	**1.00** **0.09 (0.02–0.38)**	**279/298**

Bold characters indicate association. OR (95% CI) = odds ratio (95% confidence interval), n/a = not applicable. rs312480 polymorphism was not included in the analysis because it only presented two genotypes (C/C and C/T). * Strong significance level at *p* ≤ 0.004 (after Bonferroni correction) and ^ϯ^ moderate significance level at *p* between 0.05 and 0.004.

**Table 5:** The association of CACNA1D, KCNQ1, KCNJ11, and CACNA1E haplotypes with T2DM. Data were adjusted for the covariates sex, age, and BMI.

Gene	Haplotype	Frequency	*p*	OR (95% CI)
*CACNA1D*	CGC	0.755	---	1.00
	**CAC**	**0.085**	<0.0001 *	**48.69 (12.14–195.25)**
	**CAG**	**0.073**	<0.0001 *	**0.00 (0.00–0.02)**
	**CGG**	**0.0389**	0.000004 *	**40.77 (5.00–332.33)**
*KCNQ1*	CGAC	0.6727	---	1.00
	**TTCT**	**0.294**	**0.015 ^ϯ^**	**1.85 (1.13–3.04)**
	CTCC	0.013	0.7	0.71 (0.12–4.12)
	CTAC	0.011	1.00	n/a
*KCNJ11*	GC	0.579	---	1.00
	GT	0.350	0.073	1.55 (0.96–2.49)
	AC	0.069	0.82	0.91 (0.42–1.99)
*CACNA1E*	CAG	0.394	---	1.00
	CGG	0.211	0.38	1.28 (0.74–2.23)
	TGA	0.176	0.19	0.62 (0.30–1.27)
	TAA	0.106	0.08	0.42 (0.16–1.12)
	**TGG**	**0.062**	0.0002 *	**7.14 (2.55–19.98)**
	TAG	0.022	0.15	3.41 (0.64–18.27)
	**CGA**	**0.015**	**0.023 ^ϯ^**	**13.36 (1.45–123.23)**
	CAA	0.011	0.54	2.33 (0.16–34.32)

Bold characters indicate association. OR (95% CI) = odds ratio (95% confidence interval), n/a = not applicable. * Strong significance level at *p* ≤ 0.004 (after Bonferroni correction) and ^ϯ^ moderate significance level at *p* between 0.05 and 0.004.

## Data Availability

The data that support the findings of this study are available on request from the corresponding author (R.G.-R.). The data are not publicly available because they contain information that could compromise the privacy of the patients who participated in the research.

## Supplementary Materials

The following supporting information can be downloaded at: https://www.mdpi.com/article/10.3390/ijms25179196/s1.
