# Supplementary material for: Association Study of CACNA1D, KCNJ11, KCNQ1, and CACNA1E Single-Nucleotide Polymorphisms with Type 2 Diabetes Mellitus
Source: Int J Mol Sci. 2024 Aug 24;25(17):9196. doi: 10.3390/ijms25179196 (PMC11395491; doi:10.3390/ijms25179196)

Supplementary table 1. SNPs information*

Gen	SNP	Chr	Position	Alleles	Location
CACNA1D	rs312480	3	53495113	T>C	5' UTR
	rs312486		53511427	G>A, C, T	Intron
	rs9841978		53696708	G>A	Intron
	rs12487452		53758384	C>A, G	Intron
KCNQ1	rs2283171	11	2574057	A>G, T	Intron
	rs2074196		2803570	G>A, T	Intron
	rs2237892		2818521	C>T	Intron
	rs163184		2825839	T>C, G	Intron
	rs2283228		2828300	A>C, G	Intron
	rs2237897		2837316	C>T	Intron
KCNJ11	rs5218	11	17387522	G>A, T	Exon Ala-Ala
	rs5219		17388025	T>A, C, G	Exon Lys-Stop
CACNA1E	rs10797728	1	181560616	T>A	Intron
	rs175338		181636029	A>C, G	Intron
	rs3753737		181676091	G>A	Intron
	rs2253388		181717613	T>A, C, G	Intron

* Information taken from the dbSNP database found in the NCBI (https://www.ncbi.nlm.nih.gov/snp/). Chr = Chromosome.

Supplementary table 2. Haploviews dataquality checks for the SNP

SNP No.	Name	Position	ObsHET	PredHET	HWpval	%Geno	MAF	Alleles	Rating
1	rs2283171	2574057	0.591	0.5	0.0024	100	0.492	G:A	
2	rs2074196	2803570	0.429	0.436	0.8545	100	0.321	G:T	
3	rs2237892	2818521	0.382	0.421	0.1405	100	0.301	C:T	
4	rs163184	2825839	0.342	0.495	1.12E-07	100	0.45	G:T	BAD
5	rs2283228	2828300	0.412	0.432	0.4813	100	0.316	A:C	
6	rs2237897	2837316	0.385	0.422	0.165	100	0.302	C:T	
7	rs5218	17387522	0.126	0.13	0.8585	100	0.07	G:A	
8	rs5219	17388025	0.488	0.455	0.2683	100	0.35	C:T	
9	rs312480	53495113	0.093	0.089	1	100	0.047	C:T	
10	rs312486	53511427	0.229	0.213	0.3061	100	0.121	C:G	
11	rs9841978	53696708	0.269	0.288	0.3307	100	0.174	G:A	
12	rs12487452	53758384	0.547	0.47	0.0066	99.7	0.377	C:G	
13	rs10797728	181560616	0.432	0.367	0.0026	100	0.243	A:T	
14	rs175338	181636029	0.432	0.427	0.9779	100	0.309	G:A	
15	rs3753737	181676091	0.498	0.498	1	100	0.465	A:G	
16	rs2253388	181717613	0.508	0.465	0.1384	100	0.367	C:T	

Supplementary figure 1

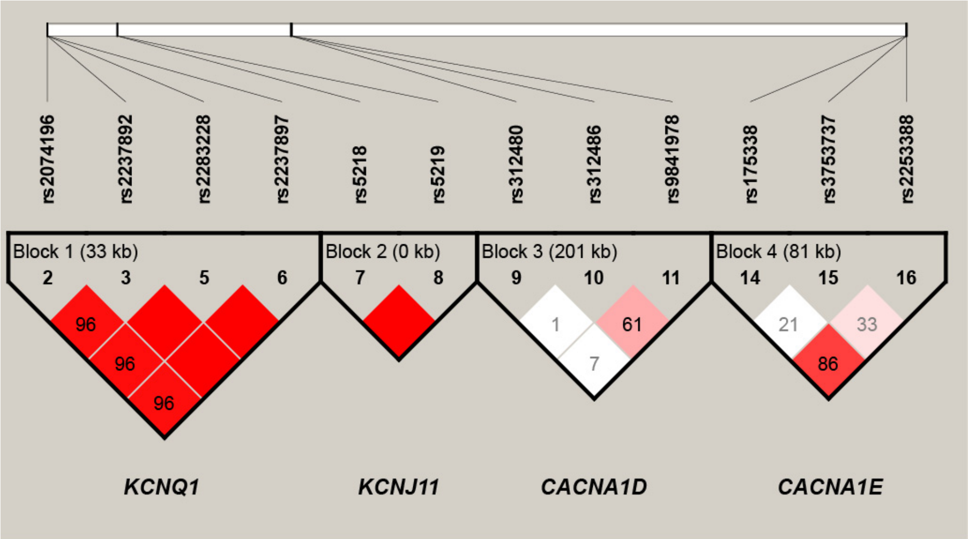

Supplement: Supplementary file 1 [file ijms-25-09196-s001.zip › ijms-3146613-supplementary.pdf]
